# The Geospatial Analysis of Patients Seeking Dental Care at a Private Dental Institution in Chennai, India

**DOI:** 10.7759/cureus.50806

**Published:** 2023-12-19

**Authors:** P Rahmath Meeral, Meignana Arumugham I

**Affiliations:** 1 Department of Public Health Dentistry, Saveetha Dental College and Hospitals, Saveetha Institute of Medical and Technical Sciences, Saveetha University, Chennai, IND

**Keywords:** patient need, chief complaint, geo mapping, dental hospitals, geospatial mapping

## Abstract

Background and aim

Assessing the priority of the patient towards dental needs while considering the distance traveled by them is important to improve a dental service. The purpose of this study was to rank the patients' felt needs for dental care in the private dental institution in order of importance. The objective is to count the number of patients with various major complaints and compare how far patients traveled for various treatment plans tailored to their individual needs in a private dental institution.

Material and methods

The sample consisted of all new patients who sought dental care at Saveetha Dental College and Hospital, Chennai, India, between January 1, 2022, and December 31, 2022. Retrospective data from the dental information archive system was gathered on their primary chief complaint and residential address. Frequency distribution of patients with different chief complaints was found. The mean and standard deviation of distance traveled by patients for different treatment plans was done using descriptive statistics using IBM SPSS Statistics for Windows, Version 22 (Released 2013; IBM Corp., Armonk, New York, United States).

Results

The priority order of the felt need by the patients was dental pain in which n=1299 (15.4%) > missing teeth with n=1224 (14.59%) > deposit/stains/halitosis n=1149 (13.6%) > shaking tooth n=936 (11.15%) > irregularly placed/proclined teeth n=852 (10.15%) > dislodged crown/restoration n=843 (10.05%) > tooth decay/discoloration n=759 (9%) > general checkup n=723 (8.6%) > swelling/ulcer/wound n=246 (2.93%) > painful jaw/facial pain/difficult mouth opening n=198 (2.26%). In 2022, most patients requiring caries preventive measure (0.8%) located at the mean distance of 10.75±2.2 km, while patients requiring scaling (16.9%), dental filling (10.9%), endodontic management (18.6%), extraction (23.7%), prosthetic replacement (13.9%), orthodontic management (10.9%), and facial pain management (2.2%) were located at the mean distance of 14.49±8.2 km, 10.28±6.25 km, 18.43±13.9 km, 14.29±6.6 km, 23.49±11.8 km, 11.76±8.13 km, and 45.32±17.35 km, respectively.

Conclusion

More number of patients traveled long distances even more than 50 km for painful tooth decay. Also, lots of patients were found to seek replacement of their missing teeth next to pain. Thus, dental pain and missing teeth form a major priority of the patient's felt dental need. Also, the patient had traveled a lot for facial pain management compared to other treatment needs which shows the lack of facial pain management practice by dental care centers near their local residence.

## Introduction

Oral diseases remain a major and growing global public health challenge, with 3.5 billion people suffering from untreated dental-related problems. Dental caries, periodontitis, and edentulousness pose the major cause of oral disease burden globally. Oral diseases are a serious impediment to the economic growth of a society and can catastrophically affect the quality of life of an individual and hence diminish his or her contribution towards the welfare of the nation [1]. To meet the global goals for oral health, more work and possibly new strategies will be required. Efforts to address socioeconomic determinants can be the basis of a conceptual five-tier public health action framework pyramid that consists of public health interventions that alter the health context (for example, consuming fewer cariogenic foods), protective interventions with long-term benefits (for example, applying pit and fissure sealants), direct clinical care (for example, the restoration of dental caries), and educational counseling at the top [2]. For this action, we felt need-based workforce planning is very important for optimal resource allocation in providing good dental care to a population at the community level [3]. Felt dental needs are those that an individual perceives for themselves and turns into the action of seeking dental care [4]. Quantifying the felt needs of patients aids in setting priorities for dental health services and research. This can be done by investigating the chief complaint of the patient recording in their own words of signs and symptoms that caused them to seek health care [5].

On the other hand, the geographical distribution of patients accessing oral health services has been examined previously in research settings to improve the extension of health care services [6].

Data on chief complaints and distance traveled by the patients to seek dental care service help us discover the treatment priorities that the population seeks. This information greatly helps public health professionals to focus more on the prioritized treatment needs for population satisfaction and create awareness about the unperceived dental needs of the population.

With this rationale, this study aimed to determine the dental patients' feelings of need and the distance they traveled to access dental care in a private dental institution. The objective is to determine the distribution of patients with different feelings of need and the distance traveled by them to private dental institutions.

## Materials and methods

The current study is a retrospective cross-sectional study that analyzed the patient data available in digital software used at Saveetha Dental College and Hospital (SDCH), Chennai, India. The ethical approval for the retrospective analysis was obtained from the institution's Scientific Review Board with the registration number SRB/SDC/PHD-2102/22/037.

The author (RM) accessed the patient data from the Dental Information Archive System (DIAS) software (http://dias.sdc.saveetha.com/). Included in the study were all patients requesting dental care at SDCH, Chennai, from January 1, 2022 to December 31, 2022. Since the dental institution hospital would be the only option accessible outside of working hours, those seeking care on weekends and/or public holidays, all patients under the age of 18, and those with unknown addresses were excluded. The data collected for the current study includes chief complaints and addresses of residents. All patient information was anonymized, but unique patient identification numbers were retained to identify patients with more than one chief complaint.

From the retrospectively collected data, all the patients were categorized into 10 groups according to their provided chief complaint, which includes a general checkup, dental deposit/stains/halitosis, tooth decay/discoloration, painful decayed tooth, shaking tooth, dislodged crown/restoration, missing tooth, irregularly placed/proclined teeth, painful jaw/facial pain/difficult mouth opening, and swelling/ulcer/any wound. Further, the patients under these categories were subcategorized according to the decided treatment plan, which includes caries preventive measures, scaling, filling, root canal treatment, extraction, prosthetic replacement, orthodontic management, and facial pain management. Also, the straight-line distances traveled by patients with different treatment plans according to their complaints were found using Google Maps.

The retrospectively collected data were analyzed using IBM SPSS Statistics for Windows, Version 22 (Released 2013; IBM Corp., Armonk, New York, United States). Descriptive statistics were done for the frequency distribution of patients with various chief complaints and various treatment needs. The mean and standard deviation of the distance traveled by patients for different treatment needs (from the treatment plan decided) were also calculated.

## Results

**Figure 1:**
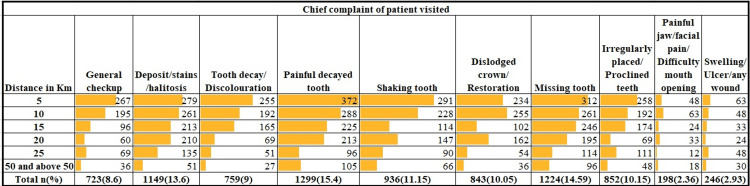
Distribution of patients with different chief complaints

The study's inclusion criteria were met by 8229 out of the 8388 individuals who sought dental care at SDCH. According to the primary complaints (felt needs) shown in Figure 1, the patients were divided into groups. With n=1299 (15.4%), it was found that the greatest proportion of patients needed therapy for painful tooth decay. Other needs felt by the patients were listed in priority order as follows: missing teeth (n=1224; 14.59%) > deposit/stains/halitosis (n=1149; 13.6%) > shaking tooth (n=936; 11.15%) > irregularly placed/proclined teeth (n=852; 10.15%) > dislodged crown/restoration (n=843; 10.05%) > tooth decay/discoloration (n=759; 9%) > general checkup (n=723; 8.6%) > swelling/ulcer/wound (n=246; 2.93%) > painful jaw/facial pain/difficult mouth opening (n=198; 2.26%).

Table 1 displays the total percentage of home distance to SDCH. In 2022, the majority of patients in need of a preventative measure for caries (0.8%) were located at a mean distance of 10.75±2.2 km, while those in need of scaling (16.9%), dental fillings (10.9%), endodontic management (18.6%), extractions (23.7%), prosthetic replacement (13.9%), orthodontic management (10.9%), and facial pain management (2.2%) were respectively located at mean distances of 14.49±8.2 km, 10.28±6.25 km, 18.43±13.9 km, 14.29±6.6 km, 23.49±11.8 km, 11.76±8.13 km, and 45.32±17.35 km.

**Table 1:** The cumulative proportion of patients with different dental treatment needs with their residential distance to SDCH SDCH: Saveetha Dental College and Hospital; km: Kilometers

Treatment plan	Total number of patients n (%)	Mean distance traveled by patients in km
For caries preventive measures	69 (0.8)	10.75±2.2
For scaling	1419 (16.9)	14.49±8.2
For filling	915 (10.9)	10.28±6.25
For root canal treatment	1563 (18.6)	18.43±13.9
For extraction	1992 (23.7)	14.29±6.9
For prosthetic replacement	1170 (13.9)	23.49±11.8
For orthodontic management	915 (10.9)	11.76±8.13
For facial pain management	186 (2.2)	45.32±17.35

**Figure 2:**
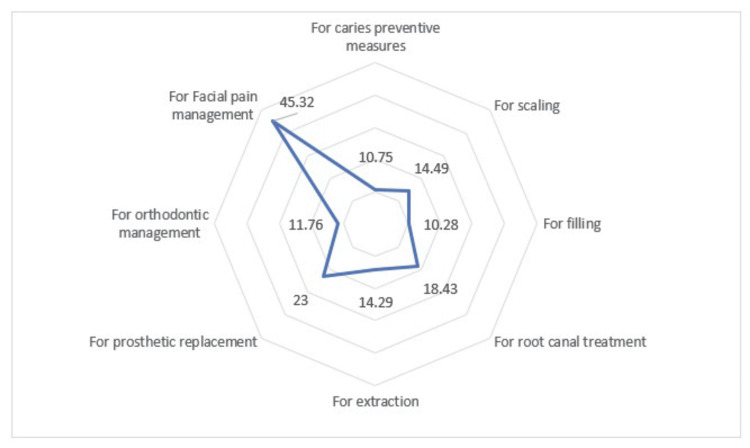
The mean distance traveled by patients for various dental treatment needs

Figure 2 depicts the average distance in kilometers traveled by patients in private dental facilities (SDCH) for various treatment plans for various felt requirements.

## Discussion

The chief complaint of the patients represents the demand for dental care [7]. The patient's primary concern is undoubtedly the issue that brought them to the dentist; if not, they will look for care elsewhere. The SDCH is located on the national highways of Chennai, in close proximity to the numerous heavily populated villages and towns. Considering the provision of comprehensive dental care to the public as its primary purpose, the current study was carried out to extend the dental service via a dental treatment camp with a mobile dental unit, along with the spreading of oral health awareness according to the patient’s felt need.

The result of the present study revealed that dental pain was the major chief complaint of the majority of patients attending private dental institutions. This was in accordance with the previous study by Maheswaran et al., in which toothache was the most common reason for demanding dental treatment [8]. The current study revealed that 16.9% of patients needed scaling, i.e., periodontal care, 18.6%, and 23.7% needed surgical therapies, i.e., root canal treatment and extraction of teeth, respectively, and 10.9% needed dental filling restorative care, which was in line with the previous study in a dental university hospital where 17% of patients needed periodontal care, 34% of the patients required surgical care, and 27% needed restorative care [9].

According to the result of this study, 9% of patients reported tooth decay as a chief complaint, which is less than the previous study in India, which had a 30% chief complaint of tooth decay [9]. This result also recommends that dental institution hospitals spread awareness to seek dental treatment for dental caries at an earlier stage before the need for surgical intervention. The lesser presentation of tooth decay as a chief complaint might be due to the prevalence of wrong myths existing among the population. Sindhu et al.'s 2020 study revealed that their study population felt “extraction of upper teeth causes loss of vision” and “cleaning the teeth loosens the teeth'' [10].

Also, 8.6% of the patients reported a general checkup of their oral cavity. A plausible explanation for this positive step by patients may be that knowledge and awareness about oral health and its effect on general well-being have increased in the population over the past few years. Only 0.8% of the patients in the current study had the opportunity to take caries preventive measures. The explanation for this may be that, due to a lack of awareness about early self-detection of dental caries, most of the patients might have developed dental caries. This result clearly highlights the importance of creating awareness about the etiology, development, early detection, and prevention of dental caries among the population, which can be achieved by carrying out various outreach programs in and around the institution. The reason for this might be attributed to the cost of preventive dental care in low-income families [11].

The chief complaint of deposits, halitosis, or stains was given by 13.6% of patients. This might be expected due to the fact that most people have low awareness about periodontal care. Apart from this, the reason for reduced felt need for dental complaints other than dental pain might be due to cost expenditure and longer treatment duration [12]. This kind of dental care neglect can be a reason for the development of destructive periodontal disease that results in tooth mobility and ultimately edentulousness. This explains the occurrence of the results of the current study related to tooth mobility and edentulousness. Shaking teeth was the chief complaint in this current study by 11.15% while missing teeth or teeth were reported by 14.59% of patients. This is in line with a previous study in Bangalore, which showed most of the patients were unaware of the existing periodontal condition in their oral cavity [13].

The concern about irregularly placed teeth was reported by 10.15% of patients. The notable occurrence of this might be due to the consideration of aesthetic profile, which has increased recently. A study in Saudi Arabia found the high aesthetic desire of patients that had driven them to get orthodontic treatment, which was in line with the results of this current study. Most of the patients (15.4%) reported a painful tooth decay complaint. This emphasizes the further need to make people aware of the importance of regular checkups and of seeking treatment early as a routine necessity for maintaining their oral health. About 10.05% of patients reported dislodged crowns. The chief complaint with difficult mouth opening, facial pain, and a painful jaw was 2.36%. Fewer patients reported complaints of ulcers, swelling, or any wound, with a statistical count of 2.93%. Correct information and knowledge about oral health are necessary to enable individuals to make appropriate decisions regarding seeking dental treatment [14].

A patient’s choice of healthcare facility is affected by geographical factors, available transportation, and proximity to his or her workplace or residence [15]. In this context, distance traveled to a dental facility refers to the patient’s preferred dental care facility and use of dental care. The result of the current study shows that the distance traveled for dental pain that led to dental filling, root canal treatment, and dental extraction was 10.28±6.25 km, 18.43±13.9 km, and 14.29±6.9 km, respectively. Also, 14.49±8.2 km were traveled for scaling treatment. This result was in line with the evaluation of barriers to dental care in the neighboring state of Andhra Pradesh by Nagarjuna et al. in 2023, which showed that 60% of the patients attending community health centers conveyed that they visit the dental clinic only in the presence of dental pain, while 26.3% of them considered the distance traveled to reach the dental clinic area to be a long one [16]. Patients in this study traveled 45.32±17.35 km for the management of facial pain, which can be explained by the fact that most of the dental care services were not focused on the proper management of temporomandibular joint disorders or neurological pain management [17]. For orthodontic treatment, 11.76±8.13 km was traveled, which shows that they neglect the distance to be traveled even though it requires frequent dental visits if they need aesthetic correction. This is in line with the results from the previous study by Shin and Cho, which showed that Korean patients traveled 1.17 times farther when compared to other dental treatments by bypassing the nearby clinics [15]. Likewise, the distance traveled for prosthetic replacement was 23.49±11.8 km, which was considerably higher when compared to the distance traveled for other treatments. This can be explained by the fact that dental replacements can be strictly done by a professional dental team, while dental pain management can be done by self-medication most of the time [18].

The findings of this study suggest that spatial distance acts as a utilization barrier and demands additional opportunity costs. At the same time, patients’ preferences for services also increase their willingness to bypass nearby dental clinics and travel greater distances. The limitation of this study is not differentiating the preference between types of population, like whether the included patients were from rural or urban areas; also, the obtained results were not compared according to the patient's socioeconomic status, which could highly influence the felt needs of patients. So the results of the current study cannot give any clear conclusion about the priority order of felt dental needs by the particular type of population. Future studies are recommended, considering socioeconomic status, to discover the preferences of different types of dental care among different socioeconomic status groups.

## Conclusions

Within the limitations of this study, the results show that dental pain is the most felt and needed by most of the patients. Also, they have neglected the distance barrier for seeking dental care management related to facial aesthetics and prosthetic replacement therapy. The lack of temporomandibular and facial pain-related management by the majority of the regional dental clinics was reflected in the higher distance traveled by patients seeking facial pain care.

Comparatively, fewer patients are seeking general checkups and preventive dental procedures, which can be escalated only through oral health promotion activities in the community. Further studies comparing the felt needs of patients with their age, socioeconomic status, rural or urban residence, knowledge, and attitude toward dental care can help in formulating customized oral health awareness programs specific to different groups of the population.
